# Intensity statistics in the presence of translational noncrystallographic symmetry

**DOI:** 10.1107/S0907444912045374

**Published:** 2013-01-16

**Authors:** Randy J. Read, Paul D. Adams, Airlie J. McCoy

**Affiliations:** a Department of Haematology, Cambridge Institute for Medical Research, Wellcome Trust/MRC Building, Hills Road, Cambridge CB2 0XY, England; b Lawrence Berkeley National Laboratory, Berkeley, CA 94720-8235, USA

**Keywords:** translational noncrystallographic symmetry, intensity statistics, twinning, maximum likelihood

## Abstract

In the case of translational noncrystallographic symmetry (tNCS), two or more copies of a component in the asymmetric unit of the crystal are present in a similar orientation. This causes systematic modulations of the reflection intensities in the diffraction pattern, leading to problems with structure determination and refinement methods that assume, either implicitly or explicitly, that the distribution of intensities is a function only of resolution. To characterize the statistical effects of tNCS accurately, it is necessary to determine the translation relating the copies, any small rotational differences in their orientations, and the size of random coordinate differences caused by conformational differences. An algorithm to estimate these parameters and refine their values against a likelihood function is presented, and it is shown that by accounting for the statistical effects of tNCS it is possible to unmask the competing statistical effects of twinning and tNCS and to more robustly assess the crystal for the presence of twinning.

## Abstract 2

The statistical effects of translational noncrystallographic symmetry can be characterized by maximizing parameters describing the noncrystallographic symmetry in a likelihood function, thereby unmasking the competing statistical effects of twinning.

## 1. Introduction

There have been great advances in the methods available for macromolecular crystallography, such that a significant fraction of structure determinations are now relatively straightforward. However, there is still the potential for serious complications when the crystals possess features that break the assumptions underlying the routine structure-solution pathways. The presence of translational noncrystallographic symmetry (tNCS) is particularly insidious in causing difficulties in all stages of crystal structure determination, from indexing the diffraction pattern to refining the structure.

In tNCS, two or more crystallographically independent copies are in the same (or nearly the same) orientation in the unit cell. Their contributions to a structure factor have the same (or similar) amplitudes but have relative phases determined by the projection of the translation vector on the diffraction vector. As a result, they interfere constructively for some reflections and destructively for others, so that there is a systematic modulation of the sum of their contributions. The most serious case is when the translation is approximately, but not exactly, equal to a potential lattice translation such as a centring operator or a cell doubling. The exact relationship is often broken by a small rotation (typically less than 10°) in addition to the translation. Such translations are referred to as pseudo-translations or pseudo-centrings because of their pseudo-crystallographic nature, and they lead to pronounced effects, with large numbers of systematically very weak and very strong reflections. The perturbation of the distribution of intensities leads to difficulties with statistical tests based on intensity statistics, as well as violating the assumptions behind likelihood targets for phasing and refinement, which assume that the data follow an isotropic Wilson distribution.

Translational NCS is a frequent issue in solved macromolecular crystal structures. The frequency of tNCS has been investigated by Zwart *et al.* (2005 ▶). The existence of tNCS can be detected by the presence of a large non-origin Patterson peak. Using the criterion that a non-origin peak greater than 20% of the origin peak was present in a Patterson map computed using data to 5 Å resolution, it was found that about 8% of structures deposited in the Protein Data Bank (PDB; Berman *et al.*, 2000 ▶) probably possess tNCS. Translational NCS can also prevent structure solution, for which there are anecdotal accounts but no statistical records.

In the following, the effect of tNCS on structure-factor intensity statistics is investigated. A method to characterize the parameters describing the tNCS has been developed and tested, and it is shown that corrected intensity statistics can be used to detect the presence of twinning. The implications for molecular replacement, experimental phasing and refinement will be explored in subsequent publications.

## 2. Statistical effects of noncrystallographic symmetry

A full maximum-likelihood treatment of NCS would cover the very general case of a number of different components that are related by different noncrystallographic symmetries. In practice, the NCS-related deviations in structure-factor intensities from an isotropic Wilson distribution are most serious when there is exact translational NCS or nearly exact translational NCS (a small rotation is present), particularly if these are translations close to crystallographic centring operators and if only one set of NCS operators is present. For this reason, and for simplicity of notation, we will only deal with the case where there is one set of NCS operators, although the formulae are presented in a way that may be generalized to multiple sets of operators. In order to deal with the very common case that the relationship is not a perfect translation but is rather a translation combined with a small rotation, we start with the case of NCS operations that combine translations with rotations of any size.

### 2.1. Covariance elements sensitive to the effects of noncrystallographic symmetry

The statistical effects of NCS are easiest to evaluate by considering correlations between NCS-related contributions to the structure factors and then assembling them into a picture of the overall effects of NCS.

As pointed out by Bricogne (1997 ▶), the presence of NCS leads to modulations in the intensities, which can be used to characterize the nature of the NCS. The following treatment of intensity statistics is similar in spirit to that of Bricogne, with the addition of an allowance for small random differences among the NCS-related copies in the positions and scattering factors of the atoms that make them up. As in Bricogne (1997 ▶) we will not consider correlations among structure factors, so the structure factors are all implicitly assumed to be for reflection **h**.

Consider a crystal containing in its asymmetric unit two or more copies of components with similar structure. The total structure factor (**F**) is made up of contributions from copies related by a combination of *N*_ncs_ noncrystallographic and *N*_sym_ crystallographic operations,
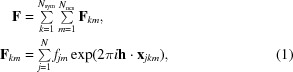
where
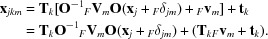



In this, there is an allowance for differences in the scattering factors for atoms in different copies (*f_jm_* could differ among NCS-related molecules *m*, particularly because of differences in the incorporated effects of *B* factors). The coordinates are represented in terms of those from a canonical copy of the molecule centred on the origin and conformational differences relative to that molecule (_*F*_δ*_jm_*). For convenience, we can take the canonical copy to be in the same orientation as the copy with *k* = *m* = 1, so that **x** *_j_* = **x**_*j*11_ − _*F*_ **v**_1_ − _*F*_δ_*j*1_ and _*F*_ **V**_1_ is an identity matrix. Note that since conformational differences are assigned even to the first copy, the canonical copy can be considered to be an average structure. The number of atoms in one copy of the component is given by *N*. The NCS rotations could be represented in terms of one matrix, **C**, in the notation used by Bricogne (1997 ▶), but the physical meaning is easier to understand in terms of rotations (_*F*_ **V**_*m*_) in orthogonal space, so that the transformations from (**O**) and to (**O** ^−1^) fractional coordinates must be included explicitly. The crystallographic symmetry operations are represented by a rotation matrix, **T** *_k_*, and a translation vector, **t** *_k_*.

We start by considering the covariances among the contributions to the structure factor where (similar to the case of experimental phasing; Read, 2003 ▶) terms between common atoms will dominate, 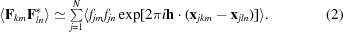



For covariances involving atoms within the same copy (*k* = *l* for crystallographic symmetry and *m* = *n* for noncrystallographic symmetry), we can consider the atoms to be independent because we have factored out any relationships leading to correlations, 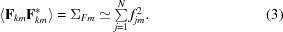



If the expressions for the transformed coordinates are entered explicitly, the dot product inside the exponential in (2) can be expanded as follows:
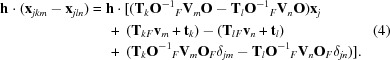



With some rearrangement and changes of variable, this can be expressed more succinctly:
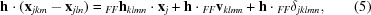
where 
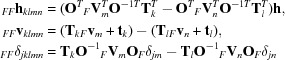
so that
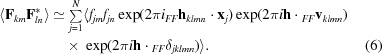



The first exponential term in (6) accounts for the effect of rotation on interference, with *_FF_* **h** *_klmn_* being equal to the difference between two copies of the original index **h** rotated by different combinations of crystallographic and noncrystallographic symmetry in the crystal; the closer *_FF_* **h** *_klmn_* is to zero, the larger the interference effect. The second exponential accounts for a systematic translation-derived phase shift between the contributions of the two copies of the component. The third exponential (along with the scattering factors) accounts for the effects of differences among the NCS-related copies. Note that if the coordinate differences are considered to be drawn randomly from a spherically symmetric distribution, then rotating these differences (*e.g.* in the variable *_F_*δ*_jm_*) will not change the nature of their probability distributions, so that the distribution of *_FF_*δ*_jklmn_*will be independent of *k* and *l*. (The subscripted prefix *FF* indicates terms relating two contributions to the observed structure factor, **F**, to distinguish them from terms relating contributions involving calculated structure factors, **G**. Such terms will be needed for subsequent work on applications to molecular replacement, experimental phasing and refinement.)

For the covariances between copies related purely by crystallographic symmetry (*m* = *n* but *k* ≠ *l*), the presence or absence of tNCS is not relevant. These terms will only differ significantly from zero when the symmetry rotation is parallel to the diffraction vector (**T** *_k_^T^* **h** = **T** *_l_^T^* **h**, so that *_FF_* **h** *_klmn_* = 0). When there is no phase shift between the contributions of these copies, they will contribute to increasing the expected intensity factor; otherwise, they will lead to systematic absences. Such pairs of contributions can be handled in a simple fashion by setting the covariance terms for *m* = *n*, *k* ≠ *l* to zero and then multiplying the remaining diagonal elements in the covariance matrix by the usual expected intensity factor ∊.

The interesting covariances are those between copies related by noncrystallographic symmetry (*m* ≠ *n*). If we assume that the differences in scattering factors and atomic positions are independent of the positions of the atoms within the components, then the expected value can be treated as a product of expected values, separating the correlation (_*FF*_ρ_*mn*_) of the structure factors for the components if they were in the same position and orientation from the interference effects, 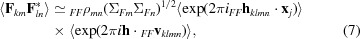
where
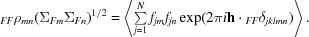



If there is an atomic model, then at least the approximate locations of the atoms in each component are known, so that the expected value of the rotational interference term can be computed. However, if we are characterizing translational NCS prior to structure solution, the best we will have is some idea of the envelope containing the component. In this case, the expected value of the interference term is an integral over the volume of the envelope (denoted *U_F_* for the volume of a unique component contributing to the structure factor **F**), which is equivalent to the Fourier transform of the envelope or a *G*-function (Rossmann & Blow, 1962 ▶). Because the envelope is finite in volume and does not possess crystallo­graphic symmetry, it is convenient to index it in terms of a diffraction vector (in units of Å^−1^),
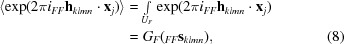
where
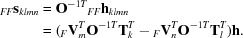



**Figure 1:**
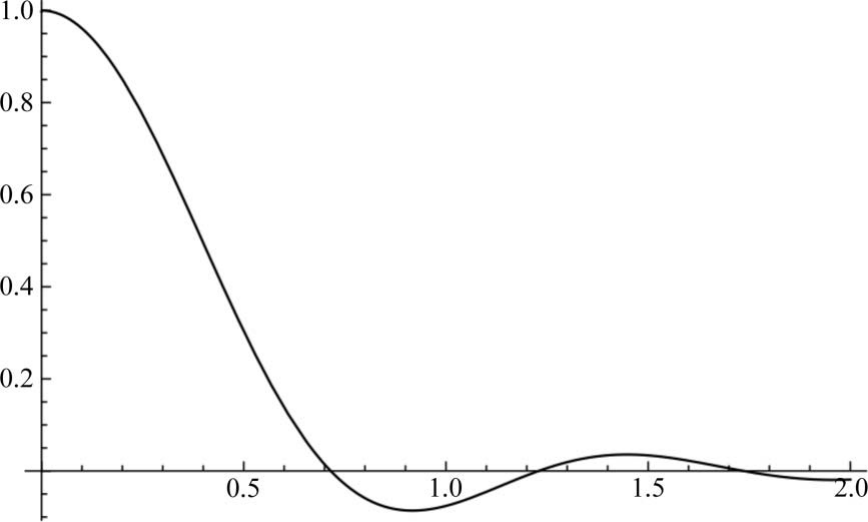
*G*-function computed from the Fourier transform of a sphere centred on the origin plotted as a function of the product of *r* and *s*, *i.e.* the ratio of the sphere radius and the resolution.

Before the shape of the molecule (or at least its orientation) is known, it may be appropriate to approximate it as a sphere with radius *r*, so that the *G*-function is the Fourier transform of a sphere (Rossmann & Blow, 1962 ▶), 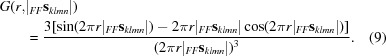



A *G*-function computed from a sphere centred on the origin (Fig. 1 ▶) gives insight into the general behaviour of the interference term; the *G*-function differs significantly from zero only for values of _*FF*_ **s**_*klmn*_ with a magnitude substantially less than the reciprocal of the sphere radius. *G*-functions from volumes with finer details in their shapes and lacking symmetry will also lack spherical symmetry and will have features extending to higher resolution, although the largest values will still be close to the origin.

The argument of the *G*-function, _*FF*_ **s**_*klmn*_, will be near zero either when the two corresponding copies of the structure component (related by combinations of crystallographic and noncrystallographic symmetry) are in nearly the same orientation or when the rotation axis is nearly parallel to the diffraction vector, so that
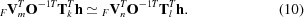



The former condition will apply for all structure factors, leading to an overall modulation of the diffraction pattern, while the latter condition will lead to spikes in the diffraction pattern with a significant modulation (Bricogne, 1997 ▶). The maximum modulation along the direction of the spikes arising from this component of the symmetry would be equal to the number of copies in the asymmetric unit. However, the maximum would only be reached if the direction of the rotation axis coincided with the diffraction vector and if the disposition of the copies were such that they were equally spaced between the Bragg planes. In principle, knowing the directions of such spikes would contribute to understanding the rotational part of the NCS, and the pattern of intensity modulation along these spikes would give information about the relative positions of copies of components. However, this is a minor contribution to the overall modulation of the structure-factor intensities in the case of translational NCS. Including this term does not significantly alter the corrective factors, but does significantly increase the computation time (results not shown). In the remainder we will neglect the contribution to the covariances of copies in significantly different orientations.

Although a noncrystallographic translation can be generated by a combination of crystallographic symmetry and noncrystallographic symmetry (for example, a crystallographic twofold and a nearly parallel noncrystallographic twofold), we can choose without loss of generality to consider the copies related by noncrystallographic translations as belonging to the same asymmetric unit, so that *k* = *l* for the pairs we will consider; the covariance elements 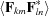 will be approximated as zero for *k* ≠ *l*. (As above, we deal with the case in which the symmetry rotation is parallel to the diffraction vector by multiplying included terms by the expected intensity factor ∊.) This leads to simplification of the expressions in the covariances, 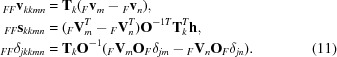



Note that the phase-shift term containing _*FF*_ **v**_*kkmn*_ now only depends on the translation vector between the NCS-related copies and not on the translational component of the crystallographic symmetry operators. This has the advantage that an analysis of the effects of tNCS can be carried out when the Laue group is known but not necessarily the particular space group.

### 2.2. Effect of tNCS on the expected intensity of the observed structure factor

Correlations among the components of the structure factor lead to systematic modulation of the observed intensities.

The variance (expected intensity) of the structure factor that is the sum of the contributions of the different components is the sum of all of the covariances between these contributions. This is simplified by the fact that we are ignoring terms between different crystallographic symmetry operators and collecting their influence in the expected intensity factor ∊. To allow simply for the possibility of a part of the crystal that does not obey these NCS operators, we can add a term Σ_*Fr*_ for the rest of the structure. (Note that Σ_*Fr*_ could include the contribution of another component with a different set of NCS operators, showing how the treatment presented here could easily be generalized.)
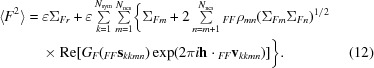



In this expression, terms with *m* < *n* have been paired with their complex conjugates, *i.e.* the terms with *m* > *n*, so that the imaginary parts cancel. The unmodulated terms can be collected into a term representing the intensity that would be expected after averaging over the modulations, Σ*_N_*, 
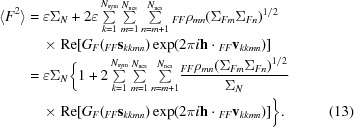



The term in the curly braces can be thought of as an extra ∊ factor accounting for the modulation of the intensities by NCS.

This general expression could be applied when there is an atomic model, which defines the envelope enclosing the parts of the structure that obey tNCS, and the rotations and translations that relate these parts of the structure. Before the structure is solved, there is no way to know the shape of the envelope (or at least how it should be oriented, if there is a molecular-replacement model), so it is simplest to assume a sphere, in which case the *G*-function is real and depends only on the resolution. This approach should capture the most important effects of tNCS even when there is a detailed atomic model, 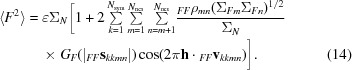



For the very common special case in which there is only one translational NCS operator, the equation can be simplified further, 
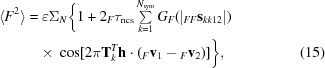
where
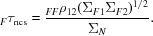



In this form, the weight _*F*_τ_ncs_ applied to the modulation term is effectively the fraction of the scattering of one component in the unit cells that obeys the translational NCS, corrected for the effect of differences among tNCS-related copies. Note that this automatically allows the presence of a component that does not obey tNCS.

## 3. Simulations to test the probability distributions

The probability distributions describing the statistical effects of tNCS have been tested by simulations in *Mathematica* (v.8.0; Wolfram Research, Champaign, Illinois, USA). In these simulations, data have been generated for a crystal in space group *P*1 containing two ‘molecules’ related by tNCS. For the first copy of the molecule, atoms were generated randomly within a sphere and copies of these atoms were then generated by applying a small rotation, a translation and a random shift. Since the molecules have a spherical envelope, the *G*-function is the Fourier transform of a sphere, as discussed by Rossmann & Blow (1962 ▶). The simulations show that accounting for the effects of orientation and conformation differences between tNCS-related copies will be essential to gain a good agreement between theory and observation.

### 3.1. Modulations of observed intensities

**Figure 2:**
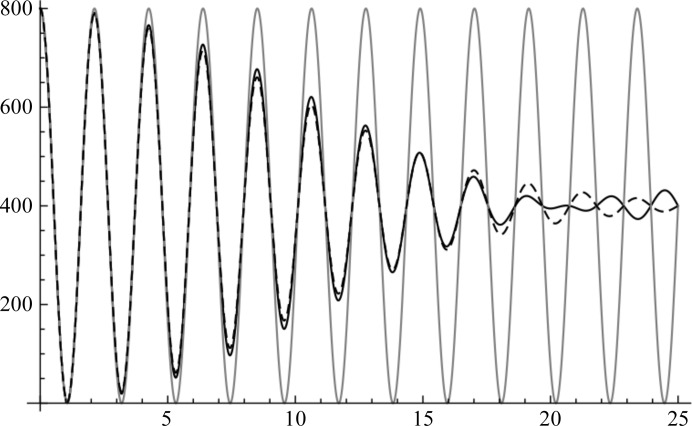
Predicted average intensity in the direction parallel to c* for a crystal (space group *P*1, unit-cell parameters *a* = *b* = *c* = 50 Å, α = β = γ = 90°) containing two copies [separated by a fractional translation of (0.47, 0.47, 0.47), *i.e.* approximately body-centred] of a spherical molecule (*r* = 20 Å) comprised of 200 single-electron point scatterers. The solid lines shows the case in which the two copies are identical in conformation but differ by a 5° rotation around the *x* axis (black line) or around the *z* axis (grey line). The dashed line shows the case in which the two copies are in the same orientation but have r.m.s. coordinate differences of 1.5 Å.

As described by (13), tNCS introduces a modulation of the expected intensities depending primarily on the phase shift of the contributions from copies related by tNCS. The modulation drops in strength if there are differences in the conformations or the orientations of the copies. Fig. 2 ▶ illustrates the effects of random coordinate differences (assumed to be drawn from a Gaussian distribution) and differences in orientation on the strength of modulation for structure factors obtained from a crystal with two spherical molecules. Note that when the model is complete and the two copies scatter with the same strength then the term _*F*_τ_ncs_ in (15) is equal to half of the complex correlation between these copies _*FF*_ρ_12_. When the coordinate differences are drawn from a Gaussian distribution with an r.m.s. coordinate difference of σ_*r*_, then this complex correlation can be calculated using the appropriate formula for σ_*A*_, which is also a complex correlation (Read, 1990 ▶),
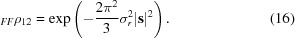



As shown in Fig. 2 ▶, random conformational differences and rotational differences between the copies can have a similar effect on the strength of the intensity modulation, except that there is a direction-dependence of the effect of the rotation difference: a rotation around the diffraction vector has no effect (because it does not change the positions of the atoms relative to the Bragg planes), whereas a rotation around an axis perpendicular to the diffraction vector has a large effect. This figure also shows that the information to distinguish the effects of random conformational differences and rotational differences may be most obvious at higher resolution.

**Figure 3:**
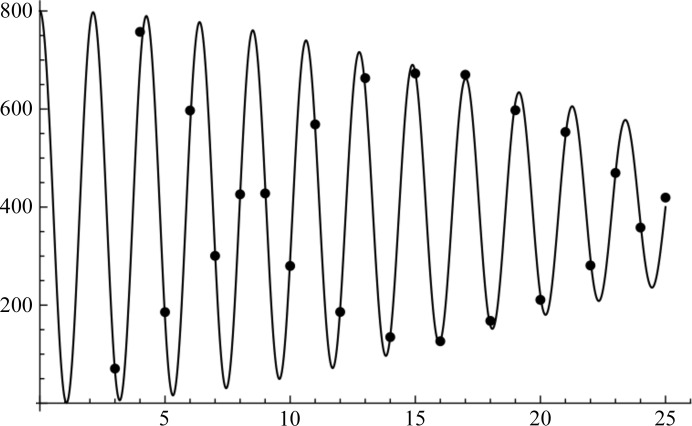
Comparison of predicted average intensity (line) with simulated average intensity (points). The crystal is equivalent to that used for Fig. 2 ▶, except that the two copies differ by a rotation of 2° around the *x* axis and an r.m.s. coordinate difference of 0.5 Å. Each point (corresponding to a 00*l* reflection) is obtained by carrying out 1000 simulations in which 200 atoms are generated randomly within the spherical envelope of the first molecule (centred on the origin); the second copy is then generated by perturbing these atomic positions followed by rotation and translation. The points for the first-order and second-order reflections are omitted because the assumptions behind the Wilson (1949 ▶) distribution are violated when the Bragg spacings are large compared with the size of the molecular envelope.

The simulation in Fig. 3 ▶ demonstrates that (15) provides an excellent description of the average intensities for different reciprocal-lattice vectors, even when there is a combination of conformational and orientation differences between the copies.

## 4. Refining parameters characterizing tNCS

To characterize tNCS from a data set, parameters describing the NCS translation, the difference in orientation of the tNCS-­related copies and the random differences between the structures of the copies must be estimated and refined. This has been implemented with the following algorithm in *Phaser* (McCoy *et al.*, 2007 ▶). The current implementation is optimized for the common case of two copies related by tNCS. Multiple tNCS copies can also be handled, as long as the copies are generated by successive applications of the same translation vector, but a more general treatment has not yet been implemented. The parameters characterizing the tNCS are refined against a likelihood function given by the Wilson (1949 ▶) distribution of amplitudes for acentric reflections,
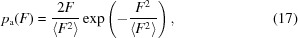
or centric reflections,
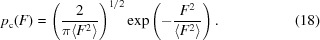



In this likelihood function, the expected value of the intensity is computed using (14), so the refined parameters are the parameters from that equation.

An initial estimate of the translation vector between the two copies (or the first two of successive copies), *_F_* **v**_1_ − *_F_* **v**_2_, is obtained from the largest off-origin peak in a native Patterson map. If the translation is close to a centring operator, symmetry-related copies of the Patterson peak will merge into a single peak on a special position. Refinement would not be able to move this translation vector to one of the equidistant symmetry copies so, if the Patterson peak is on a special position, the translation vector is first perturbed by a small translation of *d*_min_/6 in each of the *x*, *y* and *z* directions; we have found this to be sufficient to avoid the refinement being trapped on an exact centring translation.

A refinement of the relative orientation is carried out if there are two copies related by tNCS; for multiple copies, we currently approximate the effect of rotational differences as random differences among copies related by a pure translation. Because the orientation refinement does not always converge uniquely from any starting point, refinements are started from several relative orientations and that giving the best agreement with the data is chosen. The rotational difference between the two copies is parameterized as a combination of small rotations about the *x*, *y* and *z* axes, which behave well in refinement because they are approximately orthogonal. Note that when the exact shape and size of the molecule that obeys tNCS is not known, there is a trade-off between the assumed radius of the sphere that approximates the molecular envelope and the size of the rotation angles. The rotational difference enters the likelihood target through the *G*-function term, which depends on the amount by which the rotational difference rotates the diffraction vector. For small rotations, the absolute size of the movement of the diffraction vector is, to a good approximation, proportional to the rotation angle, so an error in the assumed sphere radius can be compensated by a reciprocal change in the size of the rotation angle.

Finally, the complex correlation between pairs of tNCS-related copies (_*FF*_ρ*_mn_* in 14) is currently assumed to be equivalent for all pairs when there is more than one NCS translation, and we do not currently account for the possibility of different overall *B* factors among the copies. In this case, we can refine the resolution-dependent parameter _*F*_τ_ncs_ assumed to be equivalent for all pairs of tNCS-related copies. In *Phaser* this is reported as a Luzzati *D* factor (Luzzati, 1952 ▶). In fact, the refined parameter is given by the corresponding variance term
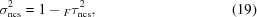
which has better refinement properties, as the likelihood function is more nearly quadratic when expressed in terms of this parameter.

## 5. Intensity moments in the presence of tNCS

Intensity moments can be a useful diagnostic for the presence of twinning (Stanley, 1972 ▶; Rees, 1980 ▶), but their usefulness can be reduced by other influences on the distribution of intensities, such as overall anisotropy and, in particular, tNCS (Padilla & Yeates, 2003 ▶; Lebedev *et al.*, 2006 ▶). Corrections for overall anisotropy are now well established (Popov & Bourenkov, 2003 ▶; McCoy *et al.*, 2007 ▶). We were interested in determining whether a further correction for the statistical effects of tNCS would at least partially unmask the statistical effects of twinning.

*E*-values that have been corrected for the statistical effects of tNCS can be computed using the expression for the expected intensity in (14),
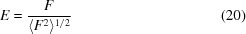
and then these *E*-values can be used in the standard moment tests.

Several test data sets were selected from the PDB for structures with pairs of molecules or assemblies in the asymmetric unit related by tNCS: PDB entries 2fuq (Shaya *et al.*, 2006 ▶), 1un7 (Vincent *et al.*, 2004 ▶), 1y9r (Fagart *et al.*, 2005 ▶), 1eh4 (Mashhoon *et al.*, 2000 ▶) and 1upp (Karkehabadi *et al.*, 2003 ▶). These cases were chosen to illustrate the effects of anisotropy, twinning and small rotational deviations from a pure translation. One of these cases, 1upp, was also chosen by Lebedev *et al.* (2006 ▶) to illustrate the effect of combining twinning and tNCS.

Table 1 ▶ shows the results that are obtained by computing second intensity moments for centric and acentric reflections before and after correction for overall anisotropy and for the effects of tNCS. Note that if the data obey standard Wilson distributions the expected value for this moment is 3 for centric reflections and 2 for acentric reflections, but in the presence of perfect twinning the moments would be reduced to 2 for centric reflections and 1.5 for acentric reflections (Stanley, 1972 ▶). To assess the significance of any deviation from the values expected for untwinned data, a *p*-value is also shown; this *p*-value is the probability (computed from the observed distribution of intensities) that the true value of the second moment for the acentric reflections is 2 or greater. In *Phaser*, a *p*-value of 0.001 or less triggers a warning that the crystal is likely to be twinned.

As an objective measure of twinning, the twin fraction obtained by twin refinement in *phenix.refine* (Afonine *et al.*, 2012 ▶) is shown for the structures in cells that support merohedral or pseudomerohedral twinning. In addition, Table 2 ▶ compares the refined values for the tNCS operators with the values determined from the deposited models to allow an assessment of the simplified model of the crystal used to characterize tNCS.

These tests demonstrate that the correction for the statistical effects of tNCS can indeed unmask the statistical effects of twinning. The *p*-values for twinned crystals are significantly lower than the threshold of 0.001 even when the twin fraction is as low as about 0.1. However, for the case of nearly perfect twinning in 1upp, the second moment is 1.71, which is significantly larger than the value of 1.5 that would be expected for perfect twinning. This may, at least in part, be because the molecular assembly differs significantly from the assumed spherical shape with a radius of about 33 Å; it is a U-shape fitting into a box of approximately 88 × 54 × 42 Å. More importantly, the twin-related reflections in this case will be affected by different modulations, so that the model of the effects of tNCS will be a compromise. In 1upp the two molecules are related by a translation of approximately 0, 1/2, 1/2 and a rotation of 3.43° about an axis very nearly parallel to the *y* axis. The largest modulations will therefore be seen for reflections with small *h* and *l* indices, for which the rotation has very little effect on scattering. However, the twin law is *k*, *h*, −*l*, so that reflections near the *h*00 axis, with large values of the *h* index and thus relatively little modulation, are superimposed on reflections near the 0*k*0 axis with significant modulation.

The results in Table 2 ▶ show that the method is able to detect deviations from exact centring operators, even when the Patterson peaks merge into a single peak consistent with a perfect centring operation. The refined translation vectors agree well with the vectors determined from the refined models. Also, even though the assumption of spherical molecules is not necessarily obeyed well, the refined rotations are correlated to the true rotations. The rotations are determined more accurately when the translations are closer to centring operators. In this situation, more of the reflections are affected by strong modulations, so that there is more signal from which the rotational parameters can be deduced.

To test whether it is important to model the rotational difference between pairs of tNCS-related molecules, or whether the refinement of the Luzzati *D* parameters can compensate, we repeated the test calculations for two of the crystals that showed a significant rotational difference, 1un7 and 1eh4, but not allowing the modelled rotation to refine away from zero. For 1un7, the mean value of the second moment of the intensity was 2.25, compared with 1.97 when the rotation was modelled. For 1eh4, the second moment without refining the rotational parameters was 1.94, compared with 1.81. Note that a second moment of 1.94 does not differ significantly from the value of 2 expected for an untwinned crystal, with a *p*-­value of 0.148. These results demonstrate that it is indeed important to model the rotational differences when characterizing tNCS.

## 6. Conclusions

This analysis has shown that the effects of tNCS depend on the exact values of the translation, which can be estimated precisely, and on small differences in orientation between the NCS-related copies, which can be given better than random estimates even under conditions where the simplifying assumptions of spherical molecules are not valid. By taking account of the statistical effects of tNCS, the statistical effects of twinning can be unmasked sufficiently to provide a clear diagnostic for twinning. This is important in practice because tests that depend on twin laws rely on having the symmetry correctly assigned (Lebedev *et al.*, 2006 ▶). If the data have been merged with too high symmetry these tests cannot be applied, but if the data have been merged with too low symmetry then these tests will generate false positives. Note that when the symmetry is correctly assigned, tests such as the *L*-test (Padilla & Yeates, 2003 ▶) are preferable for their ability to assess the twin fraction reasonably reliably. In the application of the *L*-­test, reflections with indices differing by even numbers are typically chosen to minimize the statistical effects of tNCS arising from pseudo-centring (Padilla & Yeates, 2003 ▶); however, when the tNCS differs from a pseudo-centring operation it may be helpful to correct for the statistical effects of tNCS before applying the *L*-test.

In future work, we will show how this understanding of the statistical effects of tNCS can be used to improve methods for molecular replacement, phasing by single-wavelength anomalous diffraction and structure refinement.

## Figures and Tables

**Table 1:** Second moments of intensity (〈*E* ^4^〉/〈*E* ^2^〉^2^) in the presence and absence of twinning

	Before anisotropy correction			Before tNCS correction		After tNCS correction			
PDB code	Centric	Acentric	Δ*B*_aniso_ (Å^2^)	Centric	Acentric	Centric	Acentric	Twin fraction	*p*-value
2fuq	5.04	3.10	29.0	4.42	2.81	3.01	2.01	—†	1
1un7	4.33	2.84	4.5	4.44	2.88	2.73	1.97	—†	0.221
1y9r	—	1.88	0.2	—	1.88	—	1.75	0.08	1.4 × 10^−20^
1eh4	2.45	2.35	0.0	2.45	2.38	2.56	1.81	0.10	3.6 × 10^−6^
1upp	3.05	1.84	2.3	3.04	1.84	2.52	1.71	0.46	2.1 × 10^−76^

† No merohedral or pseudomerohedral twin operator possible.

**Table 2:** Comparison of estimated and refined tNCS operators

	Rotation angle (°)			Translation vector (fractional)	
PDB code	Refined	PDB	Angular difference†	Refined	PDB‡
2fuq	0.33	0.89	0.78	−0.038, 0.497, 0.000	−0.038, 0.499, 0.000
1un7	2.05	2.52	1.05	0.487, 0.500, 0.500	0.482, 0.499, 0.500
1y9r	2.49	1.24	2.60	0.325, 0.662, 0.589	0.324, 0.662, 0.589
1eh4	3.63	4.00	1.24	0.009, 0.007, 0.493	0.002, 0.010, 0.493
1upp	4.01	3.43	2.93	0.004, −0.496, 0.494	0.007, −0.498, 0.496

† Angular difference measured using the symmetry-related transformation that agrees most closely with the NCS translation in the PDB file and choosing the (arbitrary) direction of rotation that minimizes the angular difference.

‡ PDB translation vector measured as a vector between centres of mass of common main-chain atoms
